# Contemporary survival and anticoagulation of patients with atrial fibrillation: A community based cohort study in China

**DOI:** 10.3389/fcvm.2022.911393

**Published:** 2022-07-27

**Authors:** Yong Wei, Qingye Zeng, Lidong Cai, Xingjie Wang, Bin Wang, Chaoying Shen, Chao Li, Caihong Wang, Yahong Shen, Shunhong Yang, Xiaoyu Wu, Yan Liu, Juan Xu, Xiaofeng Lu, Songwen Chen, Genqing Zhou, Shaowen Liu

**Affiliations:** ^1^Department of Cardiology, Shanghai General Hospital, Shanghai Jiao Tong University School of Medicine, Shanghai, China; ^2^Shihudang Community Health Care Center, Shanghai, China; ^3^Dongjing Community Health Care Center, Shanghai, China; ^4^Xiaokunshan Community Health Care Center, Shanghai, China; ^5^Yexie Community Health Care Center, Shanghai, China; ^6^Xinbang Community Health Care Center, Shanghai, China; ^7^Maogang Community Health Care Center, Shanghai, China; ^8^Chedun Community Health Care Center, Shanghai, China

**Keywords:** atrial fibrillation, survival, anticoagulation, mortality, stroke

## Abstract

**Backgrounds:**

The understanding of death in patients with atrial fibrillation (AF) in China is limited. This study aimed to assess the contemporary survival of AF patients in China and to explore risk factors for deaths.

**Methods:**

This was a prospective community-based cohort study including 559 AF patients, who were followed-up from July 2015 to December 2020.

**Results:**

During 66-month follow-up, there were 200 deaths (56.5% cardiovascular, 40.0% non-cardiovascular, and 3.5% unknown causes) among 559 AF patients with the median age of 76 years. The top three causes of death were heart failure (33.0%), ischemic stroke (17.0%) and cancer (16.5%). Multivariate Cox regression analysis indicated baseline variables positively associated with all-cause death were age (HR: 1.10, 95% CI: 1.08–1.13), AF subtype (HR: 1.37, 95% CI: 1.08–1.73), prior myocardial infarction (HR: 3.40, 95% CI: 1.48–7.78), previous tumor (HR: 2.61, 95% CI: 1.37–4.98), hypoglycemic therapy at baseline (HR: 1.81, 95% CI: 1.13–2.91), but body weight (HR: 0.98, 95% CI: 0.97–1.00) and use of calcium channel blocker (CCB) (HR: 0.62, 95% CI: 0.41–0.95) played a protective role to all-cause death. Of patients who were alive at the end of follow-up, 24.0% were on oral anticoagulants (OAC) alone, 4.5% on dual antithrombotic therapy, 33.1% on antiplatelet agents alone and 38.4% weren't on any antithrombotic medication.

**Conclusion:**

Ischemic stroke still remains one of the leading causes of death and OAC is seriously underused in AF patients in China. Independent risk factors for death are age, AF subtype, previous tumor, prior myocardial infarction, hypoglycemic therapy, low body weight and no CCB use.

**Clinical Trial Registration:**

http://www.chictr.org.cn/ (ChiCTR-ICR-15007036).

## Introduction

Atrial fibrillation (AF) is the most common arrhythmia for the elderly and associated with a high risk of stroke and heart failure (1). Several studies reported AF independently increased the risk of all-cause mortality by 1.5–2.0-fold (1). With the aging of population, AF has become a new global public health problem. However, great challenges exist in the prevention and treatment of AF in China, such as high morbidity, low awareness and poor management (2, 3). Both our and Du X's community-based surveys showed a great gap in anticoagulation in Chinese AF patients, for only 6.0% of them received oral anticoagulants (OAC) (2, 4).

To reduce the AF-related mortality, it is of great importance to study specific categories of deaths and to identify their risk factors for developing effective targeted interventions. However, the understanding of the mechanisms of death in patients with AF in China is limited at present. This study aimed to assess the temporal trends in survival of patients with AF in the contemporary clinical practice in China, and to identify clinical factors independently associated with deaths of specific causes. Meanwhile, to characterize the extent to which anticoagulation rate improves in Chinese community patients with AF in the latest 5 years.

## Methods

### Organization and management

In 2015, we investigated the prevalence of AF among residents over 60 years old in seven towns such as Xinbang, Chedun, Maogang, Shihudang, Dongjing, Xiaokunshan and Yexie in Shanghai China, and identified 828 AF patients from 36,734 individuals (2). Of these patients with AF, 622 agreed to receive baseline data collection and questionnaire. We informed all the 622 subjects in detail of the purpose and nature of this prospective observational study, and 90% of them signed and agreed to participate in this study. Protocols for this study are showed in Supplementary Figure 1. This study was approved by the Ethical Review Board of Shanghai General Hospital, Shanghai Jiao Tong University School of Medicine, Shanghai, China and Songjiang Central Hospital, Shanghai, China. It was in line with the declaration of Helsinki.

### Participants

The inclusion criteria were as follows: (1) aged over 60 years old, (2) registered residents in the seven above-mentioned towns, and (3) diagnosed with AF by the resting 12-lead electrocardiogram (ECG) obtained during the physical examination in 2015. Those who did not volunteer to sign an informed consent were excluded. A total of 559 AF patients were eligible and separated into paroxysmal AF, persistent AF and permanent AF groups on the basis of clinical history and previous ECG recordings in the community health care centers. All selected subjects were followed up from July 2015 to December 2020.

### Data collection

The socio-demographic characteristics [age, sex, body weight, height, body mass index (BMI)], smoking and alcohol consumption, cardiovascular disease history, other comorbidities [diabetes mellitus, stroke, tumor, chronic gastrointestinal disease (CGD), liver diseases, and renal dysfunction], drugs being used at entry, and previous intervention treatment for AF were collected at baseline for each cohort participant. The CHA_2_DS_2_-VASc score [Congestive heart failure, Hypertension, Age ≥75 years (doubled), Diabetes, Stroke/transient ischemic attack/thromboembolism (doubled), Vascular disease (prior myocardial infarction, peripheral artery disease, or aortic plaque), Age 65–75 years, Sex category (female)] was used for stroke risk stratification. The HAS-BLED (Hypertension, Abnormal renal/liver Function, Stroke, Bleeding history or predisposition, Labile international normalized ratio, elderly, Drugs/alcohol) score was calculated to estimate OAC-related bleeding risk. Smoking was defined as current smoking every day or some days and having smoked at least 100 cigarettes during the lifetime. Alcoholism was defined as self-reported drinking at least 5 days per week. Hypertension was defined as systolic blood pressure (SBP) ≥140 mmHg, diastolic blood pressure (DBP) ≥90 mmHg, or current antihypertensive therapy.

The history of heart failure was made due to the patient's symptoms, signs, previous diagnosis or treatment of heart failure, elevated brain natriuretic peptide or N-terminal pro-brain natriuretic peptide, and echocardiography.

The diagnosis of coronary heart disease (CHD) was made primarily according to clinical symptoms of angina pectoris, ECG manifestations of myocardial ischemia and coronary stenosis showed by contrast-enhanced coronary CT angiography or percutaneous coronary angiography.

Stroke was defined as a history of cerebral thromboembolism or bleeding manifested by brain computed tomography (CT) or magnetic resonance imaging. Renal dysfunction was defined as the estimated glomerular filtration rate (eGFR) <60 mL/min/1.73 m^2^ at baseline or having a history of chronic renal failure. Diabetes mellitus was defined as having a previous diagnosis of diabetes mellitus, receiving oral hypoglycemic agents or insulin treatment, or having a fasting plasma glucose ≥126 mg/dL (7.0 mmol/L) or hemoglobin A1c level ≥6.5%. Liver disease was defined as a set of chronic liver diseases (e.g., liver cirrhosis) or significant biochemical abnormalities of liver function (e.g., bilirubin more than twice the upper limit of normal, or aspartate aminotransferase/alanine aminotransferase/alkaline phosphatase over 3 times the upper limit of normal).

### Follow-up

All subjects were followed up every six months after enrollment and follow-up was censored on the date of death. The patient and family were contacted if the patient failed to return for appointments. The cause of death was independently determined by two members of the Endpoint Assessment and Adjudication Committee (EAAC) after reviewing the medical record and death certificate. Each death was attributed to a specific cause. If there was a discrepancy between the two investigators, a meeting would be held by the EAAC for discussing and voting on the cause of death. All deaths were preliminarily divided into three categories, such as cardiovascular death, non-cardiovascular death and undetermined death. Cardiovascular death was defined as any death due to cardiovascular causes (for example, myocardial infarction, heart failure, sudden death, fatal arrhythmia, pulmonary embolism, stroke). Non-cardiovascular causes of death included cancer, respiratory failure, infection/sepsis, renal failure, trauma/accidental and others. Death of unknown cause was defined as undetermined. The primary outcome of this study was all-cause death. Secondary outcomes included cause specific mortality as cardiovascular death, non-cardiovascular death and ischemic stroke-related death.

### Statistical analysis

For numerical variables, the normality test was conducted. If each group met the normality, the mean (standard deviation) was used for statistical description (BMI), and the *t-*test was performed for inter-group comparison. Otherwise, the median [interquartile interval (IQR)] was used for statistical description (age, body weight, height, SBP, DBP, HR, CHA_2_DS_2_-VASc score and HAS-BLED score), and the non-parametric test was used for inter-group comparison. Categorical data were compared between groups with Chi-Square test. And Wilcoxon rank sum test was used to compare ranked data (symptom pattern of AF and AF subtype). The relation of baseline variables with mortality was assessed using Kaplan-Meier analysis. Multivariate Cox proportional hazards models were constructed using the stepwise selection technique to identify independent predictors of deaths. All statistical analyses were performed with SPSS 13.0. Two-tailed *P* < 0.05 was considered of statistical significance.

## Results

### Baseline characteristics of participants

The media age of the 559 subjects was 76 (70–81) years and 47.9% were female. AF was paroxysmal in 18.4%, persistent in 61.2% and permanent in 20.4% of the patients.

The media CHA_2_DS_2_-VASc score was 3 (2–4), with congestive heart failure in 16.1%, hypertension in 56.2%, diabetes mellitus in 12.2%, and previous stroke in 11.1%. Detailed patient characteristics are summarized in Table 1.

**Table 1:** Patient characteristics at baseline.

**Characteristics**	**Overall** **(*****n*** = **559)**	**Alive** **(*****n*** = **359)**	**Dead** **(*****n*** = **200)**	***P*** **-value**
**Demographics**				
Age, years old, median (IQR)	76 (70–81)	74 (68–79)	81 (76–84)	<0.001
Female gender, *n* (%)	268 (47.9)	166 (46.2)	102 (51.0)	0.280
SBP, mmHg	130 (122–136)	130 (122–136)	130 (121–136)	0.764
DBP, mmHg	80(74–82)	80 (76–83)	80 (74–82)	0.163
Heart rate, bpm	80 (74–86)	78 (74–86)	80 (74–86)	0.442
Body weight, kg	60 (51–67)	62 (55–68)	55 (48–64)	<0.001
Height, cm	160 (155–166)	161 (156–168)	159 (152–164)	<0.001
BMI, kg/m^2^	23.3 ± 3.7	23.8 ± 3.6	22.4 ± 3.6	<0.001
CHA_2_DS_2_-VASc score	3 (2–4)	3 (2–4)	3 (3–4)	<0.001
HAS-BLED score	2 (1–3)	2.0 ± 1.0	2.1 ± 0.95	0.162
**Baseline lifestyle**				
Smoking, *n* (%)	173 (30.9)	113 (31.5)	60 (30.0)	0.717
Alcoholism, *n* (%)	35 (6.3)	22 (6.1)	13 (6.5)	0.862
**History of cardiovascular disease**				
Hypertension, *n* (%)	314 (56.2)	207 (57.7)	107 (53.5)	0.342
Heart failure, *n* (%)	90 (16.1)	55 (15.3)	35 (17.5)	0.502
CHD, *n* (%)	210 (37.6)	128 (35.7)	82 (41.0)	0.211
Myocardial infarction	10 (1.8)	4 (1.1)	6 (3.0)	0.201
**Comorbidities**				
Diabetes mellitus, *n* (%)	68 (12.2)	43 (12.0)	25 (12.5)	0.856
Previous stroke, *n* (%)	62 (11.1)	34 (9.5)	28 (14.0)	0.102
Tumor, *n* (%)	18 (3.2)	8 (2.2)	10 (5.0)	0.075
CGD, *n* (%)	50 (8.9)	30 (8.4)	20 (10.0)	0.514
Liver disorders, *n* (%)	9 (1.6)	4 (1.1)	5 (2.5)	0.370
Renal dysfunction, *n* (%)	11 (2.0)	5 (1.4)	6 (3.0)	0.320
Symptom pattern of AF				0.267
Obvious symptoms	78 (14.0)	52 (14.5)	26 (13.0)	
Minor symptoms	264 (47.2)	174 (48.5)	90 (45.0)	
No symptoms	217 (38.8)	133 (37.0)	84 (42.0)	
AF subtype				0.033
Paroxysmal AF	103 (18.4)	74 (20.6)	29 (14.5)	
Persistent AF	34 (61.2)	219 (61.0)	123 (61.5)	
Permanent AF	114 (20.4)	66 (18.4)	48 (24.0)	
**Treatments at baseline**				
OAC, *n* (%)	31 (5.5)	22 (6.1)	9 (4.5)	0.420
Antiplatelet agent, *n* (%)	189 (33.8)	117 (32.6)	72 (36.0)	0.414
ARB, *n* (%)	154 (27.5)	100 (27.9)	54 (27.0)	0.828
ACEI, *n* (%)	20 (3.6)	12 (3.3)	8 (4.0)	0.688
Diuretic, *n* (%)	86 (15.4)	47 (13.1)	39 (19.5)	0.044
β-blocker, *n* (%)	118 (21.1)	81 (22.6)	37 (18.5)	0.259
CCB, *n* (%)	107 (19.1)	81 (22.6)	26 (13.0)	0.006
Digoxin, *n* (%)	66 (11.8)	40 (11.1)	26 (13.0)	0.514
Statin, *n* (%)	96 (17.2)	51 (14.2)	45 (22.5)	0.013
Insulin and/or oral hypoglycemic, *n* (%)	54 (9.7)	33 (9.2)	21 (10.5)	0.616
**Previous intervention treatment for AF**				
AF ablation, *n* (%)	3 (0.5)	3 (0.8)	0 (0.0)	0.489
Pacemaker implantation, *n* (%)	16 (2.9)	9 (2.5)	7 (3.5)	0.500

*IQR, interquartile range; SBP, systolic blood pressure; DBP, diastolic blood pressure; BMI, body mass index; CHD, coronary heart disease; CGD, chronic gastrointestinal disease; AF, atrial fibrillation; ARB, angiotensin receptor blocker; ACEI, angiotensin-converting enzyme inhibitor; CCB, calcium channel blocker; OAC, oral anticoagulants*.

### Temporal survival of atrial fibrillation patients in China

**Figure 1:**
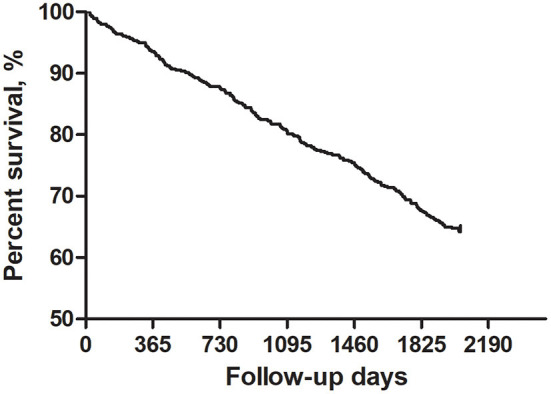
Survival of patients with atrial fibrillation.

During the follow-up, 200 patients died (35.8%). The rest 359 subjects all finished the 66-month follow-up. The survival rates from the first to the fifth year of follow-up were 93.5, 87.5, 80.5, 75.3 and 67.5%, respectively (Figure 1).

### Descriptive analysis of causes of death

A total of 200 deaths were adjudicated. The majority of deaths were cardiovascular (56.5%), whereas non-cardiovascular deaths (principally cancer) accounted for 40.0, and 3.5% of deaths were due to unknown causes. Table 2 presents death causes of the entire study population. The top three causes of death were heart failure (accounting for 33.0%), ischemic stroke (accounting for 17.0%) and cancer (accounting for 16.5%). Among cardiovascular deaths, cardiac mortality, ischemic stroke-related death and fatal cerebral hemorrhage represented 65.5, 30.1 and 4.4%, respectively.

**Table 2:** Causes of death in patients with atrial fibrillation.

**Events**	***n***	**%**
All-cause death	200	-
**Cardiovascular death**	113	56.5
Cardiac death	74	37.0
Heart failure	66	33.0
Sudden death/dysrhythmia	4	2.0
Myocardial infarction	3	1.5
Other cardiac death	1	0.5
Vascular death	39	19.5
Ischemic stroke	34	17.0
Hemorrhagic stroke	5	2.5
**Non-cardiovascular death**	80	40.0
Cancer	33	16.5
Respiratory failure	13	6.5
Infection/sepsis	12	6.0
Renal failure	2	1.0
Trauma/accidental	8	4.0
Other non-vascular death	12	6.0
**Undetermined causes**	7	3.5

### Cumulative incidences of deaths for specific causes

**Figure 2:**
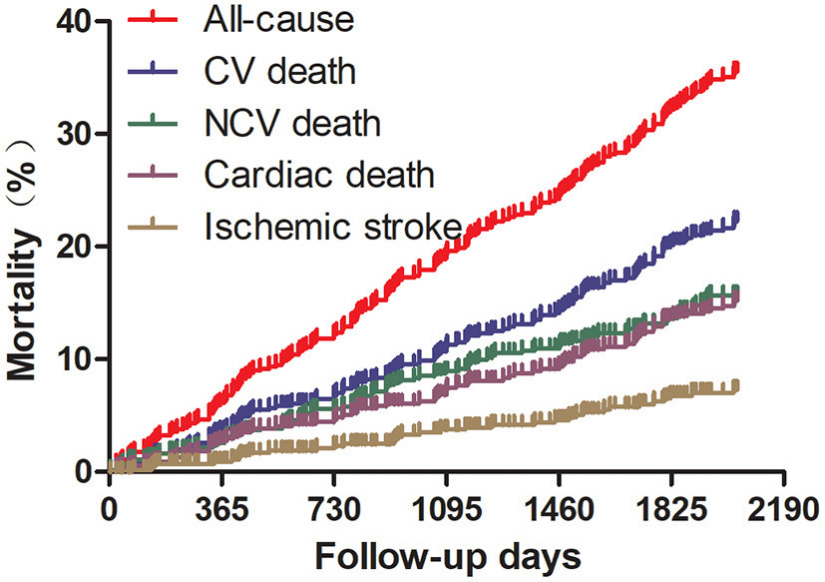
Cumulative incidences of death for specific causes. CV, cardiovascular; NCV, non-cardiovascular.

Cumulative mortality of patients with AF in the entire cohort during the follow-up is presented in Figure 2. With the follow-up of 2,562 patient-years, all-cause mortality was 7.8 per 100 person-years (%/P-Y) and the rates of cause-specific deaths were 4.4 %/P-Y for CV death, 3.1 %/P-Y for NCV death, 2.9 %/P-Y for cardiac death, and 1.3 %/P-Y for ischemic stroke-related death.

### Independent risks of death in AF patients

Among various baseline variables, only age, body weight, height, BMI, CHA_2_DS_2_-VASc score, AF type, baseline use of diuretic, CCB or statin, were of significant difference between patients who were alive at the end of follow-up and patients who had died during the follow-up (Table 1).

Multivariate Cox regression analysis indicated baseline variables associated with all-cause death independently were age (HR: 1.10, 95% CI: 1.08–1.13), AF subtype (HR: 1.37, 95% CI: 1.08–1.73), body weight (HR: 0.98, 95% CI: 0.97–1.00), prior myocardial infarction (HR: 3.40, 95% CI: 1.48–7.78), previous tumor (HR: 2.61, 95% CI: 1.37–4.98), CCB use at baseline (HR: 0.62, 95% CI: 0.41–0.95) and hypoglycemic therapy at baseline (HR: 1.81, 95% CI: 1.13–2.91) (Table 3).

**Table 3:** Independent predictors for all-cause mortality and deaths of specific causes in patients with atrial fibrillation (cox proportional hazard model, multivariate analysis).

**Baseline variables**	**HR**	**95% CI**	***P*-value**
**All-cause mortality**			
Age	1.10	1.08–1.13	0.000
AF subtype	1.37	1.08–1.73	0.010
Body weight	0.98	0.97–1.00	0.009
Prior myocardial infarction	3.40	1.48–7.78	0.004
Previous tumor	2.61	1.37–4.98	0.003
CCB use at baseline	0.62	0.41–0.95	0.027
Hypoglycemic therapy at baseline	1.81	1.13–2.91	0.014
**Cardiovascular death**			
Age	1.07	1.04–1.11	0.000
Alcoholism	2.26	1.16–4.40	0.017
Body weight	0.96	0.94–0.98	0.000
Prior myocardial infarction	5.64	2.22–14.35	0.000
CCB use at baseline	0.48	0.26–0.88	0.018
Statin use at baseline	1.59	1.02–2.48	0.041
**Non-cardiovascular death**			
Age	1.123	1.084–1.164	0.000
Diabetes mellitus	2.064	1.161–3.670	0.014
Symptom pattern of AF	1.414	1.005–1.990	0.047
AF subtype	1.619	1.101–2.380	0.014
Previous tumor	5.041	2.280–11.145	0.000
Liver disease	5.572	1.992–15.584	0.001

*AF, atrial fibrillation; CCB, calcium channel blocker*.

The strongest independent predictor of cardiovascular death was prior myocardial infarction (HR: 5.64, 95% CI: 2.22–14.35), followed by alcoholism (HR: 2.26, 95% CI: 1.16–4.40), statin use at baseline (HR: 1.59, 95% CI: 1.02–2.48), age (HR: 1.07, 95% CI: 1.04–1.11), body weight (HR: 0.96, 95% CI: 0.94–0.98) and CCB use at baseline (HR: 0.48, 95% CI: 0.26–0.88) (Table 3). Meanwhile, age, diabetes mellitus, symptom pattern of AF, AF subtype, previous tumor, liver diseases were found to be independently associated with non-cardiovascular death (Table 3).

### Current status of OAC use among AF patients alive at the end of the follow-up

**Figure 3:**
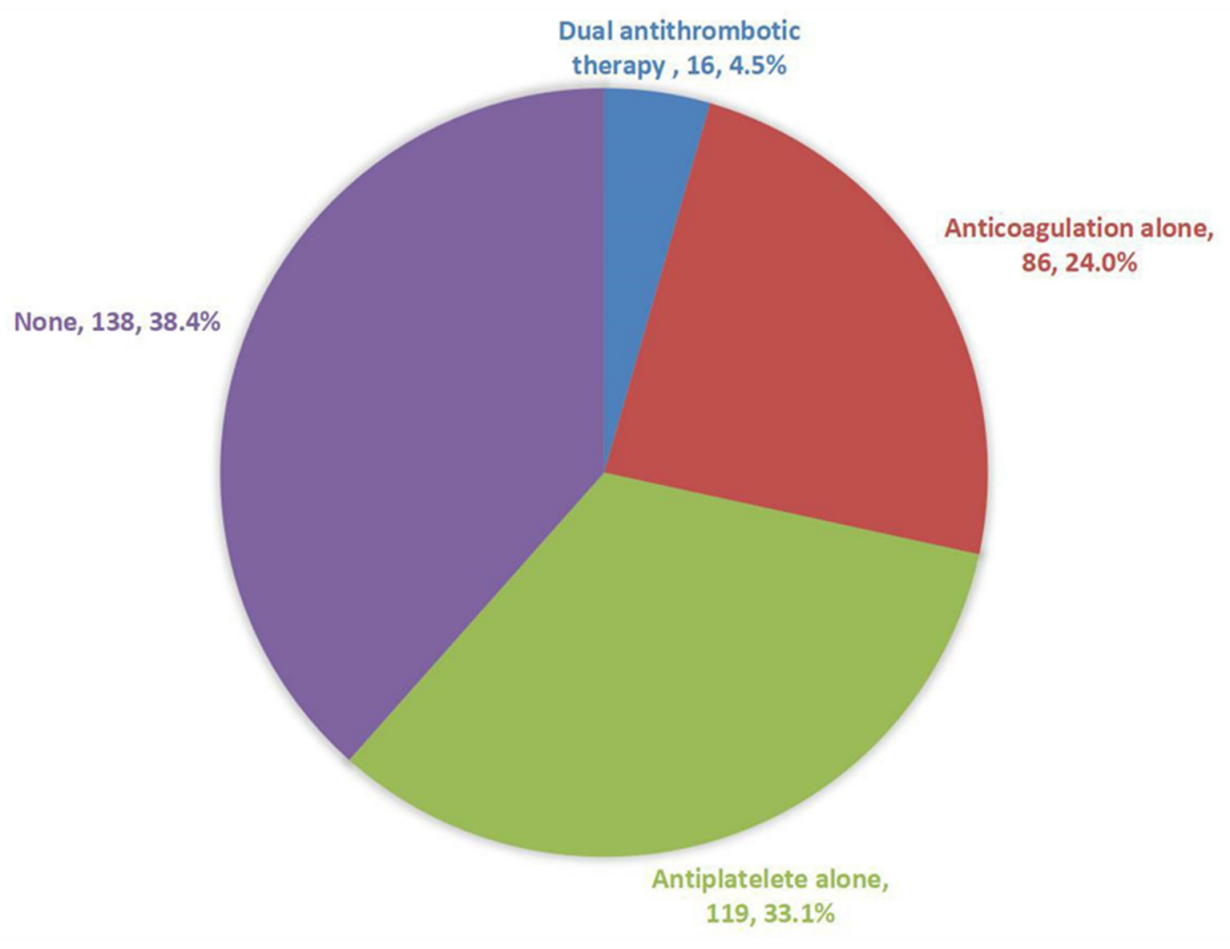
Current status of antithrombotic treatment in patients with atrial fibrillation who were alive at the end of the follow-up.

Of patients who were alive at the end of follow-up, 4.5% were on dual antithrombotic therapy (OAC plus antiplatelet agents), 24.0% on OAC alone, 33.1% on antiplatelet agents (most aspirin), and 38.4% were not on any antithrombotic medication (Figure 3). The rate of anticoagulation was 28.5%. Of patients who received anticoagulants, warfarin was prescribed in 83.3% and non-vitamin K antagonist oral anticoagulants (NOAC) in 16.7% (dabigatran in 6.9% and rivaroxaban in 9.8%).

## Discussion

The principal findings of this study were as follows: (1) the underutilization of anticoagulants of these eligible high-risk patients was observed in this community-based AF cohort in China; (2) the predominant cause of death is cardiovascular in the elderly community patients with AF in China, and ischemic stroke still remains one of the leading causes of death; (3) independent risk factors for death in these AF patients include age, AF subtype, low body weight, previous tumor, prior myocardial infarction, CCB use and hypoglycemic therapy at baseline; and (4) anticoagulantion therapy for AF patients from Chinese communities has improved in the recent 5 years, but it's still far from the recommendations of AF management guidelines.

### Mortality of AF patients

AF is independently associated with a twofold increased risk of all-cause mortality in women and a 1.5-fold increase in men (1). All-cause mortality and cause-specific deaths among AF patients have been examined in both randomized control trials and cohort studies. In contemporary anticoagulated AF population, the average annual mortality rates reported in four randomized control trials (RCTs) were 4.38 %/P-Y for the RE-LY study (5), 5.42 %/P-Y for the ROCKET AF study (6), 3.82 %/P-Y for ARISTOLE study (7) and 4.99 %/P-Y for the ENGAGE AF-TIMI 48 study (8). Although significant heterogeneity existed across these studies, a meta-analysis indicated their adjusted mortality rate was 4.72%/year (9). The real-world cohort studies indicated a great variation in mortality of AF patients, with 3.83 %/P-Y in a global hospital-based AF cohort (GARFIELD-AF) (10), 5.5 %/P-Y r in a Japanese community-based AF cohort (Fushimi AF Registry) (11), 6.3 %/P-Y in a nationwide population-based study in Korea (12), and 16.48 %/P-Y in the largest and comprehensive study on AF from India (KERALA-AF Registry) (13). Data describing the mortality of AF patients in China are limited. To our knowledge, this is the first prospective community-based study to investigate mortality of AF patients in China, with a long-term follow-up over 5 years. We found all-cause mortality in our study population was 7.8 %/P-Y. Mortality rates varied widely among different studies due to differences in study design, population, region, patient characteristics, enrollment period, health management level, and treatment option, etc.

### Causes of death in AF patients

Our data indicated cardiovascular events were the most common cause of death, accounting for 56.5% of all deaths. Such proportion was similar to a large retrospective, real-world study of hospitalized patients with AF (14), which indicated 54% were cardiovascular and 43% were non-cardiovascular among all deaths. Both RCTs (5–8) and real-world cohorts (10) indicated stroke-related death represented no more than 7% of the overall mortality. However, in our study, the death due to ischemic stroke accounted for 17.0% of all deaths. This proportion was much higher than that in the previously published reports. The only plausible explanation is that only 6% of the enrolled AF patients were treated with OAC at the baseline in our study. The significance of OAC for AF patients with high risk of stroke is evident. Underuse of anticoagulants might cause high rate of AF-related stroke and stroke-related death. This result emphasizes that it's still of great significance to develop targeted approaches to improve anticoagulation rate for further reducing mortality in Chinese AF population.

### Independent risk factors for death in AF patients

Various covariables are reported to be associated with the risk of death in AF patients. This study indicated the independent predictors of all-cause death were age, AF subtype, low body weight, previous tumor, prior myocardial infarction, CCB use and hypoglycemic therapy at baseline. Prior myocardial infarction was identified as the strongest indicator of all-cause death and cardiovascular death, which was consistent with the sub-analysis from the RE-LY trial (5). Previous studies indicated pre-existing heart failure was associated with an increased risk of all-cause death and cardiovascular death (5–7, 10), but this study didn't show such association. One potential explanation for this different finding is that patients were diagnosed with heart failure if they had a history of heart failure in the present study, regardless of heart function classification. So, many AF patients with pre-existing heart failure might have received optimal treatment of heart failure, resulting in an improved prognosis.

It's controversial whether sustained AF was associated with higher mortality than paroxysmal AF (10, 11, 14, 15). This study indicated permanent AF was independently associated with an increased risk of all-causeortality and non-cardiovascular mortality, but not cardiovascular mortality. Such correlation needs more research to verify in the future. Some studies indicated OAC use was independently associated with a lower risk of all-cause mortality and cardiovascular mortality (10, 11, 14, 15). However, our study did not show this correlation, and it may be attributed to the very low rate of anticoagulant therapy at baseline. The Fushimi AF registry demonstrated that statin use was associated with better all-cause mortality (11). However, on the contrary, we found that statin use at baseline was positively (HR: 1.59, 95% CI: 1.02–2.48) associated with cardiovascular death. Patients using statins in China usually have severe dyslipidemia or atherosclerotic cardiovascular disease, which are well-known risk factors for cardiovascular death. In addition, we also found that patients who died during follow-up had a significantly lower rate of prescription of CCB. Of note, CCB use was independently associated with a decrease of 38% in the risk of overall death and 52% in the risk of cardiovascular death in our studied patients. It's well-known that CCB is not recommended to patients with heart failure and the elderly are prone to heart failure, so elderly patients using CCB may not have heart failure, selecting a population with good prognosis.

### Current status of OAC use among AF patients in China

Although anticoagulation is recommended by all guidelines of AF management to reduce the risk of AF-related stroke, both our and other studies indicated OAC remained seriously underused in AF patients in China (2, 4). This study indicated the baseline anticoagulation rate of the studied population was only 6% in 2015, which was consistent with the previous community-based study on anticoagulation status of AF patients in China (4). Meanwhile, hospital-based studies indicated the rate of OAC use were 35.6% in Jiangshu in 2017 (16), 11.5% in Chongqing in 2013 (17), 28.8% in Xinjiang in 2015 (18) and 31.7% nationwide in 2012 (19) in China. Though great variations of OAC treatment in Chinese patients with AF were explored among different studies, the anticoagulation rate of community patients with AF tended to be much lower than that of out-patients or in-patients with AF in China. To our opinion, the community-based study can better reflect the real-world anticoagulation status of AF patients than the hospital-based study.

Several studies have forecasted a growing improvement of OAC treatment for hospitalized or outpatient patients with AF in China (20, 21). This study first described contemporary anticoagulation in AF patients in Chinese communities. The anticoagulation rate of these studied AF patients increased from 6% in 2015 to 28.5% in 2020. So, application of anticoagulants according to the AF management guidelines is still far from expected in China. It's essential to develop target measures to improve clinicians' compliance with AF management guidelines as well as AF patients' adherence to OAC. Before the onset of NOAC in China, many physicians were unwilling to prescribe warfarin to AF patients mainly for their excessive worry about bleeding and patients' poor adherence to INR monitoring in China (22). Though NOAC is currently available in China and preferentially recommended to AF patients when compared with warfarin, high cost is the main reason for limiting its use, especially for AF patients in the impoverished areas in China. In order to reduce the financial burden of AF patients, more efforts should be paid to reduce the selling price of NOAC in China and increase the proportion of expenses paid by the China Medical Insurance Fund to over 90%. Left atrial appendage occlusion (LAAO) is an alternative treatment for preventing stroke. However, this cohort showed no one had received LAAO before enrollment, and none of them received LAAO during follow-up. Not only low anticoagulation rate, but also less willing to undergo catheter ablation in AF patients in China. Our data showed only 3 cases undergoing catheter ablation during the follow-up, of which one case recurred.

## Conclusion

In conclusion, the predominant cause of death is cardiovascular and ischemic stroke still remains one of the leading causes of death for community patients with AF in China. The independent predictors of all-cause death included age, AF subtype, low body weight, previous tumor, prior myocardial infarction, CCB use and hypoglycemic therapy at baseline. Distinct clinical factors are associated with cardiovascular and non-cardiovascular deaths. OAC is still seriously underused in the contemporary clinical practice in China in spite of a growing improvement. These findings provide important information for the optimal management of AF patients in China.

## Data availability statement

The original contributions presented in the study are included in the article/Supplementary Material, further inquiries can be directed to the corresponding authors.

## Ethics statement

The studies involving human participants were reviewed and approved by Ethical Review Board of Shanghai General Hospital, Shanghai Jiao Tong University School of Medicine, Shanghai, China and Songjiang Central Hospital, Shanghai, China. The patients/participants provided their written informed consent to participate in this study.

## Author contributions

YW: had full access to all of the data in the study and takes responsibility for the integrity of the data and the accuracy of the data analysis. SL and SC: concept and design. YL, JX, LC, XWa, BW, CS, CL, CW, YS, and SY: acquisition and analysis, or interpretation of data. SL: critical revision of the manuscript for important intellectual content. GZ and XWu: statistical analysis. XL: administrative and technical, or material support. All authors contributed to the article and approved the submitted version.

## Funding

This study was supported by General Program of the National Natural Science Foundation of China (Grant No. 81970273) and Youth Program of National Natural Science Foundation of China (Grant No. 81300137 and 82000312).

## Conflict of interest

The authors declare that the research was conducted in the absence of any commercial or financial relationships that could be construed as a potential conflict of interest.

The handling editor declared a past co-authorship with one of the authors SL.

## Publisher's note

All claims expressed in this article are solely those of the authors and do not necessarily represent those of their affiliated organizations, or those of the publisher, the editors and the reviewers. Any product that may be evaluated in this article, or claim that may be made by its manufacturer, is not guaranteed or endorsed by the publisher.

## Supplementary material

Supplementary Figure 1 Study protocols.
