# Supplementary figures and images for: Contemporary survival and anticoagulation of patients with atrial fibrillation: A community based cohort study in China
Source: Front Cardiovasc Med. 2022 Jul 27;9:911393. doi: 10.3389/fcvm.2022.911393 (PMC9363600; doi:10.3389/fcvm.2022.911393)

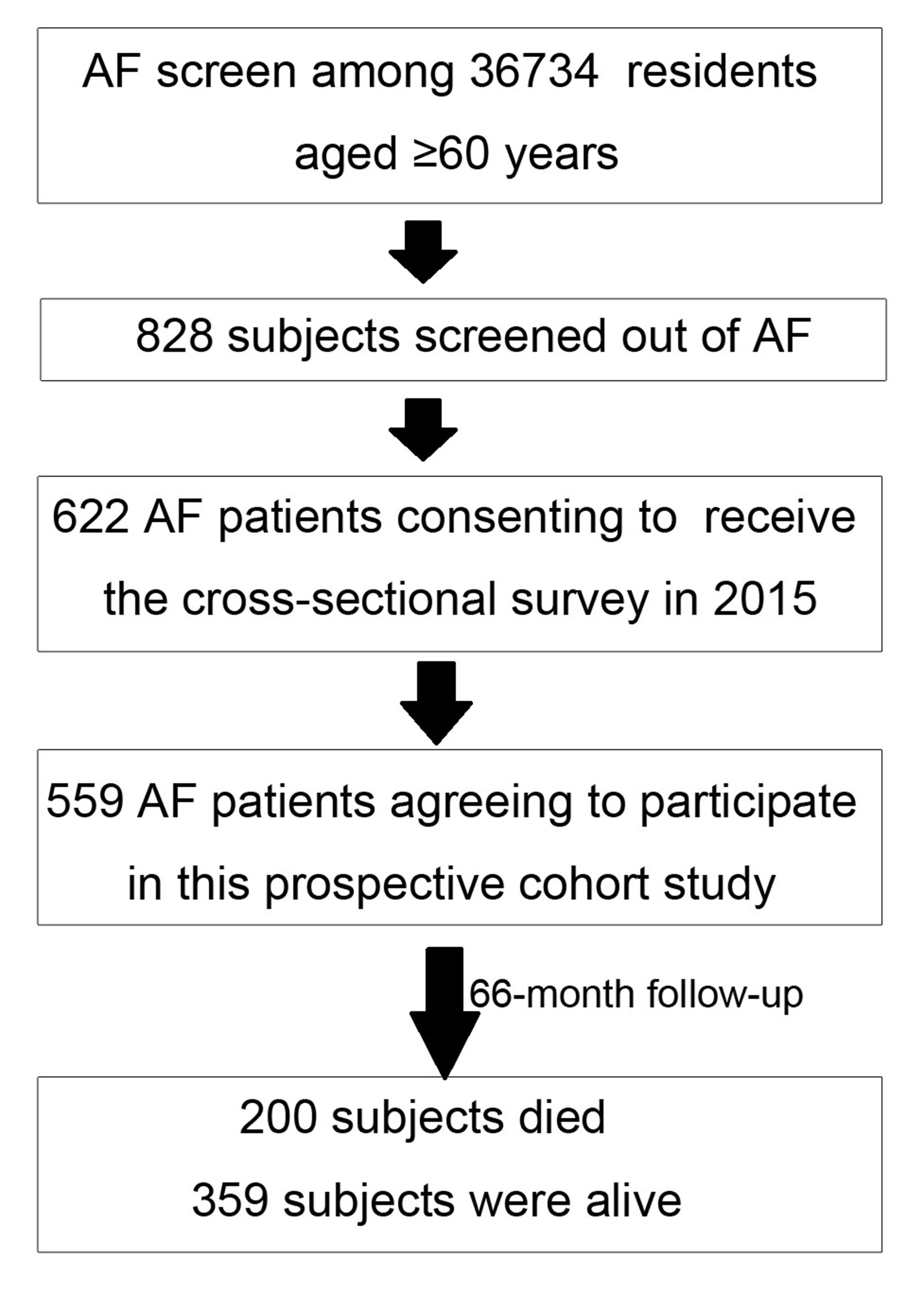

Supplement: Supplementary Figure 1 — Study protocols. [file Image_1.JPEG]
